# Outlier Handling Strategy of Ensembled-Based Sequential Convolutional Neural Networks for Sleep Stage Classification

**DOI:** 10.3390/bioengineering11121226

**Published:** 2024-12-04

**Authors:** Wei Zhou, Hangyu Zhu, Wei Chen, Chen Chen, Jun Xu

**Affiliations:** 1 Jiangsu Key Laboratory of Intelligent Medical Image Computing, Nanjing 210044, China; wei.zhou@nuist.edu.cn; 2 School of Future Technology, Nanjing University of Information Science and Technology, Nanjing 210044, China; 3 Center for Intelligent Medical Electronics (CIME), School of Information Science and Engineering, Fudan University, Shanghai 200433, China; hyzhu20@fudan.edu.cn; 4 School of Biomedical Engineering, The University of Sydney, Sydney, NSW 2006, Australia; wei.chenbme@sydney.edu.au; 5 Center for Medical Research and Innovation, Shanghai Pudong Hosptial, Fudan University Pudong Medical Center, Shanghai 201203, China; 6 Human Phenome Institute, Fudan University, Shanghai 200438, China

**Keywords:** sleep staging, deep learning, electroencephalograph

## Abstract

The pivotal role of sleep has led to extensive research endeavors aimed at automatic sleep stage classification. However, existing methods perform poorly when classifying small groups or individuals, and these results are often considered outliers in terms of overall performance. These outliers may introduce bias during model training, adversely affecting feature selection and diminishing model performance. To address the above issues, this paper proposes an ensemble-based sequential convolutional neural network (E-SCNN) that incorporates a clustering module and neural networks. E-SCNN effectively ensembles machine learning and deep learning techniques to minimize outliers, thereby enhancing model robustness at the individual level. Specifically, the clustering module categorizes individuals based on similarities in feature distribution and assigns personalized weights accordingly. Subsequently, by combining these tailored weights with the robust feature extraction capabilities of convolutional neural networks, the model generates more accurate sleep stage classifications. The proposed model was verified on two public datasets, and experimental results demonstrate that the proposed method obtains overall accuracies of 84.8% on the Sleep-EDF Expanded dataset and 85.5% on the MASS dataset. E-SCNN can alleviate the outlier problem, which is important for improving sleep quality monitoring for individuals.

## 1. Introduction

Sleep is a crucial physiological process for maintaining a healthy life [1,2]. However, in recent years, sleep disorders have caused a considerable impact on human health and garnered widespread social concern [3]. A lack of sleep can lead to a series of diseases, such as high blood pressure, heart disease, etc., that seriously affect human health [4]. Sleep staging is a common approach to diagnosing sleep quality [5]. Physicians utilize clinical guidelines and their medical expertise to classify physiological signals gathered through polysomnography (PSG), including electroencephalography (EEG), electro-oculography (EOG), and electromyography (EMG), into distinct sleep stages: wakefulness (Wake), non-rapid eye movement (NREM), and rapid eye movement (REM) [6]. The classification process ensures a systematic and accurate assessment of sleep architecture. NREM can be further divided into four stages—S1, S2, S3, and S4—according to the R&K manual [7] or into three stages—N1, N2, and N3—according to the American Medical Sleep Association (AASM) manual [8]. In clinical practice, sleep staging is usually performed manually by an experienced physician. This process is time-consuming and laborious. It may also result in subjective errors in judgment due to physician fatigue. To reduce the burden on physicians and to ensure accurate and objective assessment, numerous studies have proposed automatic sleep staging methods.

In recent years, significant advancements have been made in automatic sleep staging methods [9,10,11,12,13]. Initially, studies relied on manual features as prior knowledge, such as mean, power spectral density, Hjorth complexity, etc. [14,15,16]. These features were then combined with traditional machine learning classifiers like random forest [17], support vector machine [18], K-nearest neighbor [19], etc., for sleep stage classification. While these methods have yielded positive results, they rely on prior medical knowledge for effective feature extraction [20]. Moreover, the use of features based on prior medical knowledge is subjective, which can impact the classification effectiveness of the classifier. Recent studies have focused on utilizing neural networks for automatic feature extraction and classification learning from sleep signals, reducing the reliance on prior medical knowledge [21,22]. These neural network-based approaches have also achieved good sleep staging results [23,24]. In addition to single-structured neural networks, such as convolutional neural networks (CNNs) and recurrent neural networks (RNNs), some hierarchical networks have greatly contributed to the development of automatic sleep staging [25,26,27]. The combination of CNN and RNN architectures makes full use of the feature extraction capability of CNNs and the temporal learning capability of RNNs [28,29,30]. The combination of RNN and RNN architectures integrates both short-term and long-term temporal information present in the sleep signals [31,32,33,34]. The combination of CNN and transformer architectures is optimized based on the CNN+RNN architecture to reduce the computational overhead and improve the accuracy of sleep staging [35,36,37]. These advancements in automatic sleep staging methods have the potential to enhance the accuracy and efficiency of sleep stage classification, reducing the burden on medical professionals and providing more objective assessments of sleep quality.

However, note that both features extracted with prior medical knowledge and features automatically extracted using neural networks tend to be relatively generalized features. These features work well for sleep staging on numerous groups but sometimes perform poorly in sleep staging results for a few groups or individuals. These poorly performing sleep staging results are often considered outliers in automatic sleep staging results [38]. The presence of such outliers can significantly impact the overall accuracy of sleep staging and compromise its reliability. Furthermore, the existence of outliers can introduce biases in the feature selection process during model training. The outliers may influence the selection and weighting of certain features, potentially leading to suboptimal performance in sleep staging. Poorly assigned sleep stages can lead to erroneous sleep scoring, misdiagnosis of sleep disorders, and inappropriate treatment recommendations. These errors may prevent individuals from receiving the necessary interventions to improve their sleep quality and overall well-being. Therefore, addressing the issue of outliers is crucial to improve the robustness and accuracy of automatic sleep staging methods.

To solve the above problem, a deep learning architecture based on ensemble learning, E-SCNN, is proposed. E-SCNN exploits a priori knowledge to cluster and classify the population and uses an ensemble learning approach to weight the sleep staging results, dedicated to reducing the occurrence of outliers. E-SCNN consists of a clustering module and a sequential convolutional neural network (SCNN). In the SCNN, a sequence-encoding process (SEP) is designed to pre-process the inputs, while a sequence-decoding process (SDP) is used the post-process the outputs. The clustering module can divide each individual into subgroups that are most similar to its feature distribution, then assign weights according to the clustering distance. The SEP can expand the individual sleep stage inputs to sequential sleep stage inputs. Combined with the robust ability of the SCNN to simultaneously learn features at different scales within the input sleep stage and contextual information between sleep stages, the network can fully extract feature information from the input signal. Finally, the SDP performs a weighted averaging of the sleep staging results in multiple sequences to enhance the robustness of the model. We conducted sleep staging experiments on two datasets, namely the Sleep-EDF Expanded and MASS dataset. The results show that the clustering module led to an increase in the overall accuracy of the model from 84.4% to 84.8% on the Sleep-EDF Expanded dataset and from 85.2% to 85.5% on the MASS dataset. Meanwhile, the performance of the proposed method is close to or even better than that of state-of-the-art methods. The main contributions of this paper are summarized as follows:

1. In this paper, a model oriented to solve the problem of sleep staging result outliers within a population, namely E-SCNN, is proposed. E-SCNN clusters individuals into subgroups based on the similarity of their feature distributions, allowing for the assignment of personalized weights through an ensemble learning approach. This method significantly reduces the impact of outliers on overall sleep staging outcomes and lays the foundation for a personalized sleep staging model.
2. The convolutional kernel design of the SCNN allows for the simultaneous learning of multi-scale features within sleep stages and contextual information between sleep stages. It allows the model to capture diverse sleep-related features with low computational overhead and fast operation.
3. Unlike many studies that separate traditional machine learning and deep learning approaches, our model combines the strengths of both, leveraging their respective advantages to improve sleep staging accuracy.

The remainder of this article is organized as follows. Section 2 shows the details of E-SCNN. Section 3 describes the detailed procedure and preparation of the experiment. The results of E-SCNN are also described in detail in Section 3. In Section 4, the results are discussed from various perspectives. Finally, the conclusion of this paper are presented in Section 5.

## 2. Methods

**Figure 1:**
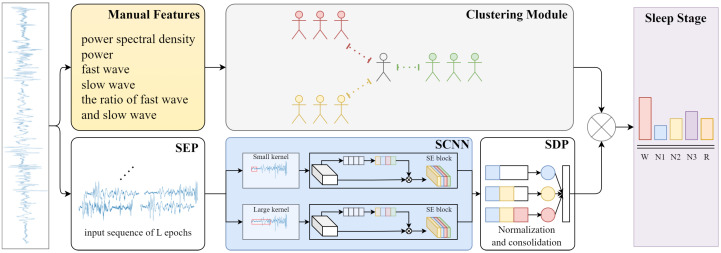
The overall flowchart of E-SCNN. First, the individual sleep signals are fed into the clustering module for detailed sub-population analysis. Second, according to the number of cluster centers, several SCNN models are trained specifically using the corresponding subgroups. The input epochs are expanded by the SEP before they are sent into the SCNN. The SDP can normalize and consolidate the probability values across different epochs generated by the SCNN. Finally, the sleep results are assigned weights by the clustering module, and the final sleep stages are outputted.

In this section, we propose a model called E-SCNN to address the problem of outliers in sleep staging. Figure 1 shows the overall structure of E-SCNN. The following subsections describe the structure and functions of each module in detail.

### 2.1. Clustering Module

The sleep characteristics of each individual are unique. Sleep characteristics may differ significantly between people [39,40,41]. The clustering module aims to divide subgroups of the population that share similar sleep characteristics. This can minimize the appearance of outliers in the sleep staging results due to differences in sleep characteristics. The specific steps of clustering are outlined as follows.

First, assume that the input signal is $X\in R^{P\times M}$, where P is the number of sleep stages, i.e., epochs, and M is the duration of each epoch multiplied by the corresponding sampling, while the features are identified by feature extraction based on some prior knowledge.

(1) $$F=FE\left(X\right)\in R^{T}$$

where FE(·) is feature extraction and T is the number of the features. These features are personalized with individual differences, such as power spectral density and the energy in different frequency bands [20]. In this paper, the power spectral density and power of the δ (0.5–4 Hz), θ (4–8 Hz), α (8–13 Hz), β (13–30 Hz), and γ (30–35 Hz) bands are extracted, as well as the ratio of fast to slow waves.

Secondly, the extracted features are subjected to principal component analysis (PCA) to reduce the redundant information of the features.

(2) $$F_{PCA}=PCA\left(F\right)\in R^{U}$$

where U is the number of principal components. The retention of information (ROI) can be calculated using the following formula:

(3) $$ROI=\frac{\sum_{i=1}^{U}\lambda_{i}}{\sum_{j=1}^{n}\lambda_{j}}$$

where $\lambda_{i}$ is the first U largest eigenvalues, $\lambda_{j}$ is all eigenvalues (there are n in total), and n is the number of features in the original data. This ratio represents the proportion of the total variance explained by the selected principal components and, therefore, reflects the degree of ROI.

Thirdly, the reduced-dimensional features are clustered in an unsupervised manner based on Euclidean distances to delineated subgroups.

(4) $$D_{C}=\frac{1}{N_{C}}\sqrt{\sum_{i=1}^{N_{C}}\sum_{k=1}^{U}{\left[f_{pca_{k}}-f_{pca_{k}}^{i}\right]}^{2}}\;C=1,2,3,\ldots$$

where C represents the subgroups, $D_{C}$ is the mean Euclidean distance between the subject and subjects class C, $N_{C}$ is the number of subjects in class C, and $f_{pca_{k}}^{i}$ represents the features of the i-th individual. In addition, the number of delineated subgroups is regulated with silhouette coefficients.

(5) $$S=\frac{1}{N}\sum_{i=1}^{N}\frac{b(i)-a(i)}{\max\{a(i),b(i)\}}$$

(6) $$a\left(i\right)=\frac{1}{N}\sum_{i\neq j}^{N_{c}}\sqrt{\sum_{k=1}^{N}{\left[f_{pca_{k}}^{i}-f_{pca_{k}}^{j}\right]}^{2}}\;\left(i,j\in C\right)$$

(7) $$b\left(i\right)=\frac{1}{N}\sum_{j}^{N_{c}}\sqrt{\sum_{k=1}^{N}{\left[f_{pca_{k}}^{i}-f_{pca_{k}}^{j}\right]}^{2}}\;\left(i\notin C,j\in C\right)$$

where S represents the silhouette coefficients, a(i) is the intra-class distance of the i-th subject from other subjects in the same class, and b(i) is the distance between the class of the i-th subject and other subjects not in the same class. The silhouette coefficients allow the optimal number of subgroups within the group to be divided. The larger the value of the silhouette coefficient, the better the effect of clustering. When an individual is validated for sleep staging, the clustering module can assign weights to the final sleep staging results by calculating the distance between each subgroups. Thus, assigning greater sleep staging weights to groups that share more similar sleep characteristics can be effective in avoiding extreme results.

### 2.2. Sequence-Encoding and Decoding Processes

Typically, the entire sleep cycle is divided into a number of 30 s epochs. To capture both spatial and temporal features within and between sleep epochs, the SEP is used to encode multiple adjacent epochs into a vector. After extracting the features via the SCNN, the SDP is employed to convert the output of the vector into a prediction for a single epoch.

#### 2.2.1. Sequence-Encoding Process

**Figure 2:**
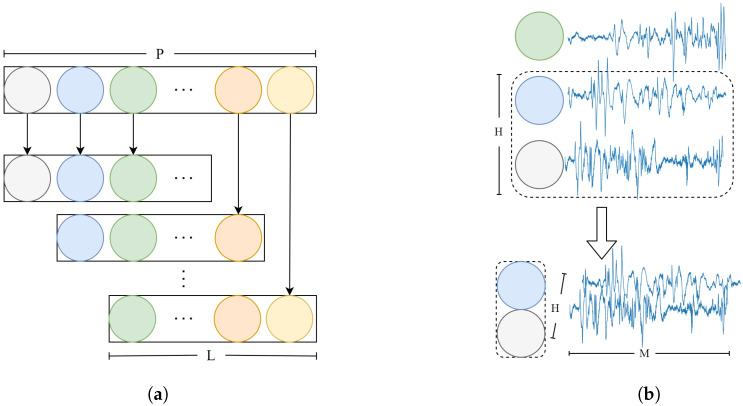
The SEP. (**a**) The transition from the input signal to sequences according to the sliding window (L), where P is the number of epochs. Each circle represents an epoch. (**b**) For each epoch, the signal is stacked with that of the adjacent epoch. H represents the selected epochs, and M is the length of each epoch. In subsequent processing, features across epochs are extracted using a 2D convolutional neural network (H = 2).

The sequence-encoding process is demonstrated in Figure 2. For the input ($X\in R^{P\times M}$, where the P is the number of epochs and M is the the sampling rate multiplied by the epoch duration), a window of length L is used to intercept the input EEG signal with a sliding window of one epoch (L = 10). The window length is selected based on insights from previous research [31]. Then, a signal ($\hat{X}\in R^{(P-L+1)\times L\times M}$) is obtained. With the sliding window, the input signal is expanded from a two-dimensional shape of P×M to a three-dimensional shape of (P−L+1)×L×M.

Through the expansion of input, the signal sent to the model undergoes a transition from separated epochs to a sequence of L epochs. In addition, information from multiple neighboring epochs is fused, thereby enhancing the sleep stage classification of the current epoch. For each epoch, the signal is stacked with that of the adjacent epoch to form a two-dimensional array. In subsequent processing, features across epochs are extracted using a 2D convolutional neural network. As shown in Figure 2b, H represents the selected length of epochs. The expansion enables the model to process temporal information and capture the intricate relationships between consecutive epochs.

#### 2.2.2. Sequence-Decoding Process

**Figure 3:**
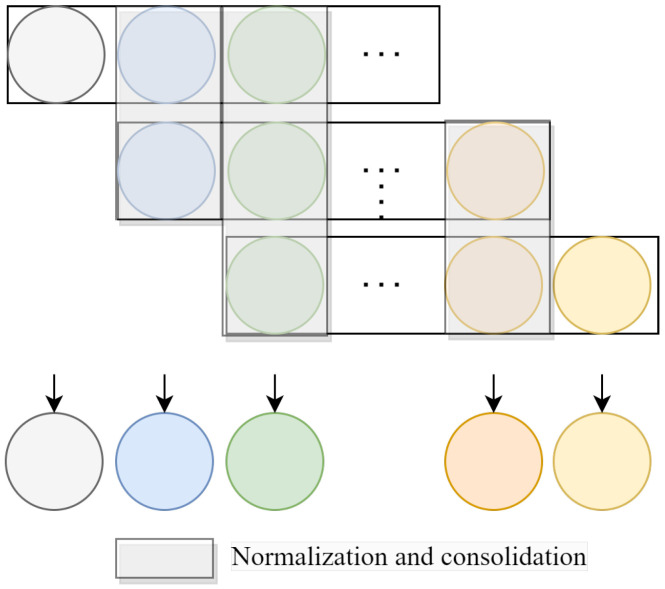
For duplicate predictions for overlapping epoch regions, the probabilities belonging to the same epoch are normalized and superimposed.

In this work, signals from multiple contiguous adjacent epochs are used to train the model. On the one hand, to fully utilize the information across epochs, a sliding window, as well as 2D convolution, is used to capture the temporal features in the SEP. On the other hand, multiple consecutive signals are utilized to predict the sleep stage of the current epoch. As shown in Figure 3, there are duplicate predictions for overlapping epoch regions. This redundancy can lead to errors, particularly when different contextual information from various sequences causes variations in the classification of a specific sleep stage. Simple probability consolidation may lead to individual differences due to varying probability values in different individuals. We propose a scheme for normalizing the probabilities before summation. The normalization process standardizes the probability values across different epochs, ensuring more consistent and reliable results.

The steps of the SEP can be summarized as normalization and consolidation. First, the probabilities of all sleep stages corresponding to each epoch are normalized. Then comes the consolidation of the corresponding predictions. Finally, the sleep stage of current epoch can be obtained by selecting the maximum value for each epoch.

(8) $${\hat{Y}}_{ij}=Norm\left(Y_{ij}\right)\;1⩽i⩽P-L+1,1⩽j⩽L$$

(9) $${\dot{Y}}_{m}=\sum_{i+j-1=m}{\hat{Y}}_{ij}\;1⩽m⩽P$$

(10) $${\ddot{Y}}_{m}=argmax\left({\dot{Y}}_{m}\right)$$

where $Y\in R^{(P-L+1)\times L\times C}$ is the probability of each sleep stage for each epoch and C is the number of sleep stages. $\hat{Y}$ is the normalized probability, and ${\dot{Y}}_{m}\in R^{P\times C}$ is the probability superimposed on the predictions of multiple epochs. The corresponding category (${\ddot{Y}}_{m}$) for each epoch can be obtained by calculating the probabilities of the different sleep stages.

### 2.3. Sequential Convolutional Neural Network

**Figure 4:**
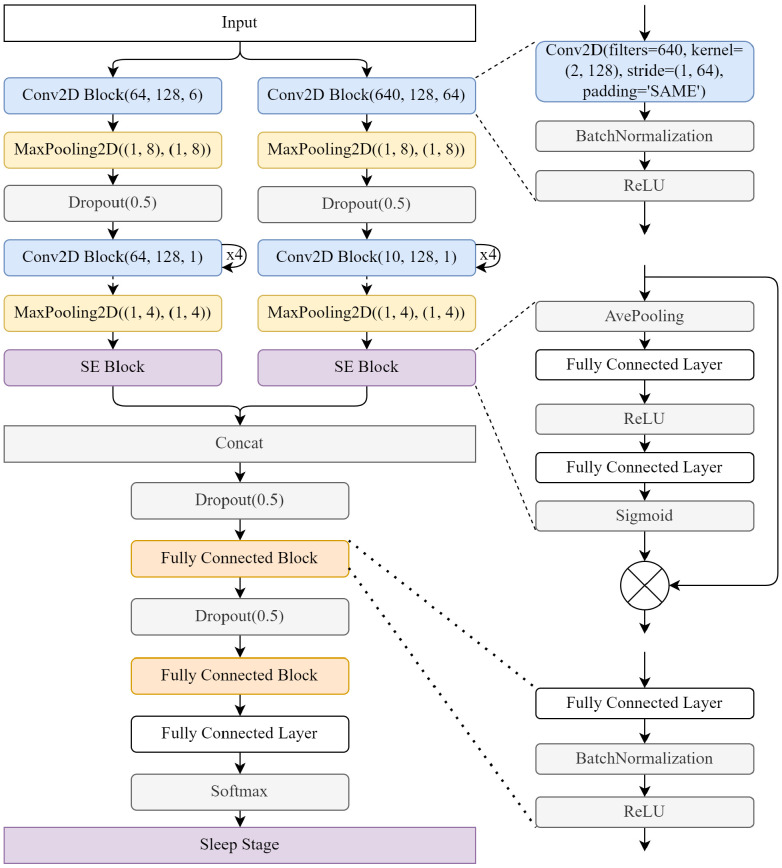
The details of the SCNN.

Figure 4 show the details of the SCNN, which incorporates two scales of CNNs with different sizes of convolutional kernels to extract high-frequency and low-frequency features from EEG signals. Specifically, small-scale CNNs with small convolutional kernels process a few data points each batch, corresponding to a small time scale and a high-frequency scale. Large-scale CNNs with large convolutional kernels process more data in each batch, corresponding to a large time scale and a low-frequency scale. Furthermore, the SCNN leverages special convolutional kernels to extract temporal information between adjacent epochs. In previous studies, the convolutional kernel height (H) was limited to 1, since the inputs consisted solely of individual epochs. However, through the sequence expansion facilitated by the SEP, the SCNN’s convolutional kernel height (H) can be set to 2 or more, allowing it to learn temporal dependencies and information between adjacent or even interdependent epochs. For this study, a convolutional kernel height of 2 is adopted for the SCNN to extract temporal information between adjacent epochs. In summary, the SCNN employs different scales of CNNs with varying kernel sizes to extract frequency-specific features from EEG signals, while the SCNN takes advantage of special convolutional kernels to capture temporal dependencies between adjacent epochs, enabling more comprehensive feature extraction for accurate sleep staging. The effectiveness and redundancy of features play crucial roles in both the training results and the efficiency of the model [42]. To enhance the model’s performance, it is beneficial to increase the weights of effective features while reducing the weights of redundant features. The Squeeze-and-Excitation (SE) block proposed by Hu et al. [43] provides a mechanism for dynamically reassigning feature weights, thereby optimizing the feature representation.

Assume that the input to the SE block corresponds to the output of one branch of the SCNN, denoted as $\dot{X}\in R^{P\times (T-L+1)\times O}$, where *O* represents the extracted features from one branch of the SCNN. The SE block employs global pooling to reduce the dimensionality of features, transforming the input ($\dot{X}$) to $\ddot{X}\in R^{P\times 1\times O}$. Subsequently, two fully connected (FC) layers are utilized, followed by a ReLU layer, to parameterize the pass selection mechanism. The mechanism reinforces the importance of center features while attenuating the significance of edge features. Then, the feature weights are activated and selected through the sigmoid layer. Finally, the weights are multiplied by the original features to obtain the reassigned features. The process can be represented using the following equation:

(11) $$\overset{˘}{X}=\dot{X}\times \sigma \left(F2\left(ReLU\left(F1\left(AvgPooling\left(\dot{X}\right)\right)\right)\right)\right)$$

where AvgPooling(·) is the average global pooling, F1(·) is the first FC layer, F2(·) is the second FC layer, ReLU(·) is the ReLU activation function, and σ(·) is the sigmoid activation function. Feature optimization of the SE block can greatly reduce the impact of redundant features on model learning and improve the efficiency and performance of the model [44]. Finally, the features of the two scales are concatenated to obtain the feature ($F_{st}\in R^{T\times L\times U}$, where *T* is the batch size, *L* is the length of the sequence, and *U* is the size of concatenated features).

(12) $$F_{st}=concat\left({\overset{˘}{X}}_{large-scale},\;{\overset{˘}{X}}_{small-scale}\right)$$

By incorporating the SE block, the model can dynamically adjust the feature weights, promoting the discriminative power of important features while suppressing less informative ones. This adaptive feature reassignment process contributes to the optimization of the model’s performance in the sleep staging task.

### 2.4. Ensemble Learning

In this work, we propose an ensemble strategy to combine machine learning and deep learning. The clustering module and classifier (SCNN) are trained separately using different features, then ensembled in a weighted-voting manner. Weights are computed based on the distance of sample points from the clustering center, combined with the probabilities predicted by multiple SCNN models to facilitate voting. The ultimate sleep stage result is an adaptive fusion of predictions derived from diverse architectural approaches. Initially, cluster analysis is adopted to divide the population into subgroups. Depending on the number of subgroups, SCNN models are trained separately for the population. The final result is determined by the weighted fusion of the predictive probabilities of multiple SCNN models and the corresponding distances of the samples to the clustering centers. The fusion process is illustrated as follows. First, the distances of each sample to the different clustering centers are calculated. Second, the probability predicted by the SCNN for each epoch is obtained. There are as many SCNN models as there are clustering centers. Finally, the probability of each epoch belonging to a particular sleep stage is computed by incorporating the normalized probability, weighted by the associated distance.

(13) $$y_{pred}=argmax\left(\sum_{i=1}^{C}{\dot{Y}}_{i}*\frac{1}{D_{i}^{2}/\sum D_{i}^{2}}\right)$$

where ${\dot{Y}}_{i}$ is the superimposed probability obtained by the i-th SCNN, as shown in Equation (9); $D_{i}$ is the distance to the clustering centers in Equation (5); and *C* represents the number of clustering centers.

## 3. Results

In this section, we describe the data used in the experiment, the pre-processing performed on the data, and the evaluation metrics. After that, we show the results of our experiments in detail, including the effects of the parameters in the clustering module, the sleep staging performance of the ensemble learning model, and a comparison with existing methods.

### 3.1. Dataset

Two public datasets were used to validate model performance. The datasets are described in Table 1.

#### 3.1.1. Sleep-EDF Expanded

The Sleep-EDF Expanded dataset encompasses two distinct subsets: the sleep cassette (SC) and sleep telemetry (ST) subsets, compiled between 1989 and 1994 [45]. In this paper, the SC subset was utilized, comprising recordings from 78 healthy participants aged between 25 and 101 years old. Each PSG recording within the SC subset consisted of a horizontal EOG channel, two channels of Fpz-Cz and Pz-Oz EEG, and an EMG channel. The EEG signals were sampled at a frequency of 100 Hz. The sleep stage annotations were manually assigned in accordance with the R&K standard and subsequently adjusted to comply with the AASM standard.

#### 3.1.2. Montreal Archive of Sleep Studies (MASS)

MASS was collected from different hospitals [46], involving whole-night sleep recordings of 200 subjects (97 females and 103 males) aged from 18 to 76 years old. The MASS consisra of five subsets, SS1–SS5, which were manually annotated based on the AASM standard or the R&K standard. In this experiment, we used the SS3 subset. The MASS SS3 dataset includes 62 healthy subjects, comprising 29 males (age 40.4 ± 19.4) and 33 females (age 44.2 ± 18.6). The details of the selected EEG channel and samples of different sleep stages are shown in Table 1.

To reduce the influence of potential interference present in the PSG signals during sleep staging, a series of preprocessing measures were implemented. First, all signals were filtered through a notch filter set at either 50 or 60 Hz, effectively removing the artifacts. Additionally, a bandpass filter with a frequency range spanning from 0.3 to 35 Hz was applied to further filter the irrelevant frequency components. Furthermore, to minimize the impact of inter-individual variations, all the PSG signals were normalized to a zero mean and a standard deviation of one. The preprocessing was designed to enhance the quality and consistency of the PSG signals, consequently leading to more reliable sleep staging results.

### 3.2. Experimental Setup

The performance of the model was validated utilizing the leave-one-subject-out (LOSO) technique, which entails designating a single subject as the test set while training and validating the model on the remaining subject data. It can provide a comprehensive assessment of the model’s performance and exhibit sensitivity to outliers. In order to assess the capability of the model, a wide spectrum of conventional evaluation metrics were utilized. Specifically, accuracy (Acc) provides a straightforward ratio of correctly predicted instances among total predictions. The F1 score (F1) adeptly balances precision and recall, making it particularly advantageous in scenarios characterized by lopsided data distributions across multiple classes. Cohen’s Kappa (κ) offers a nuanced perspective by measuring the degree of agreement beyond chance in classifying samples. Moreover, the specificity (Spec) and sensitivity (Sens) metrics delve into the model’s capacity to accurately distinguish between positive and negative outcomes, respectively. This comprehensive suite of metrics collectively illuminates the overall performance.

### 3.3. The Performance of the Clustering Module

In this paper, thirteen features, such as the power spectral density, power, and fast and slow waves, were extracted. To reduce the computational complexity and increase the efficiency of the model, PCA was used for feature optimization. Table 2 shows the relationship between the number of components and the amount of information retained after PCA, as well as the silhouette coefficients corresponding to the different numbers of subgroups after clustering.

**Figure 5:**
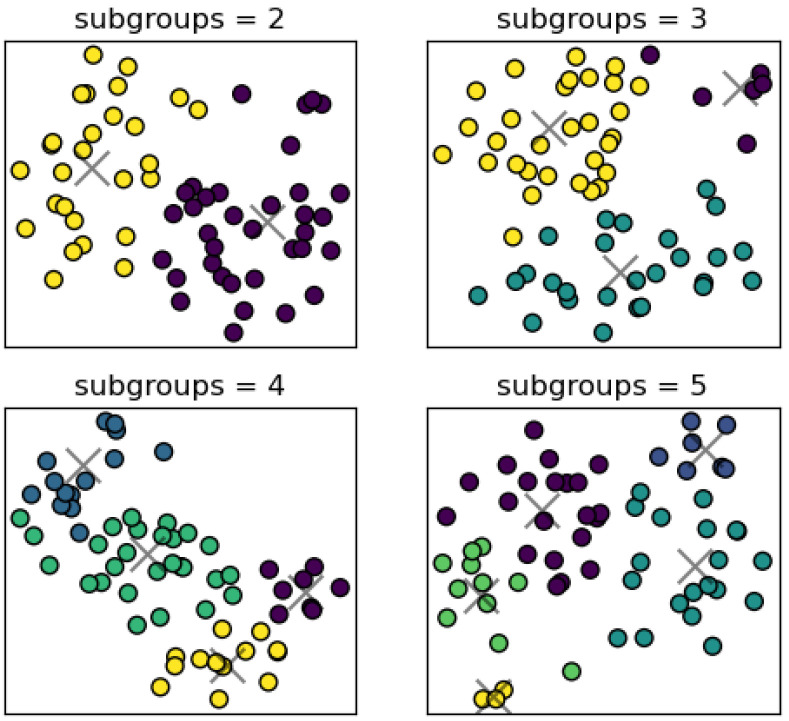
T-SNE plot of extracted features and the center of clustering on the MASS dataset.

As shown in Table 2, for different numbers of principal components, the silhouette coefficient was generally higher when the number of subgroups was two. Simultaneously, it exhibited a fluctuating trend as the number of clustering subgroups increased. It was noteworthy that the highest silhouette coefficient (36.1%) was found with two subgroups and the number of principal components was three, which may imply that the subjects were more easily classified into two subgroups at lower dimensions. Figure 5 reflects the T-Distribution Stochastic Neighbor Embedding (t-SNE) plot for different settings on the MASS dataset. It was obvious that the features were most pronounced when divided into two groups. Therefore, the number of principal components was set to three and the number of subgroups was set appropriately as two.

### 3.4. Overall Performance of the Proposed Method

Table 3 shows the performance of E-SCNN. For the SC dataset, the SCNN model achieved an overall accuracy of 84.4% and a κ of 83.8%. The model performed particularly well on the REM stage, with an F1 score of 87.0%.

When E-SCNN was applied to the same dataset, there was a slight improvement in both accuracy and κ, with values of 84.9% and 84.3%, respectively. The F1 score also slightly increased to 78.1%, indicating a slight improvement in overall performance across all stages. The results on the MASS dataset also highlight the effectiveness of E-SCNN compared to the SCNN model.

**Figure 6:**
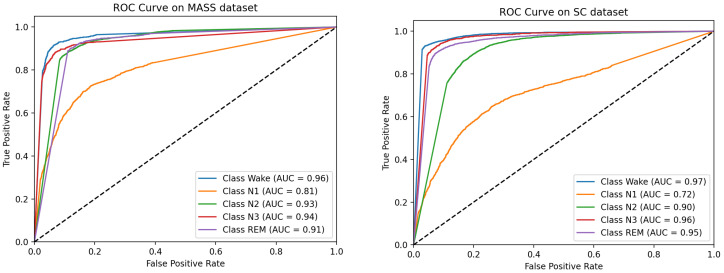
ROC curve of E-SCNN on two datasets.

Figure 6 demonstrates the receiver operating characteristic (ROC) curve of E-SCNN, in which the area under the curve (AUC) reflects different aspects of classifier performance. In the context of multiclass classification problems, a common strategy to obtain individual ROC curves for each sleep stage involves treating each sleep stage as positive and the others as negative. As shown in Figure 6, the AUC essentially reflects the trade-off between the true-positive rate (TPR) and the false-positive rate (FPR) for each sleep stage. The results are consistent with those presented in Table 3, with the model performing poorly in the N1 stage and with high reliability in the other stages.

### 3.5. Comparison with State-of-the-Art Methods

**Figure 7:**
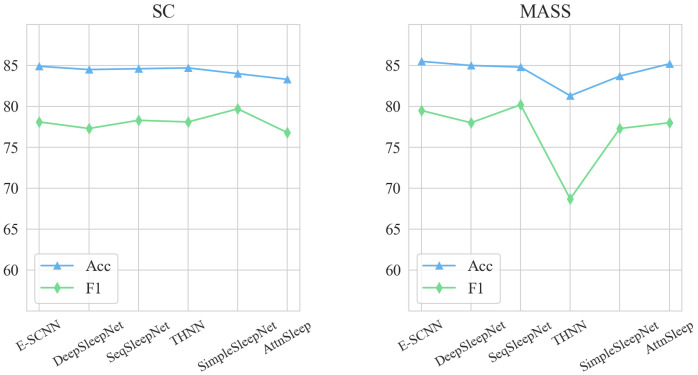
Accuracy and F1 comparison of different methods on two datasets.

Table 4 compares the the results of the proposed method with those of other state-of-the-art methods. These methods encompass architectures such as a CNN combined with LSTM, Seq2Seq frameworks, and hybrid models integrating CNNs with transformer architectures. We benchmarked against a variety of highly influential approaches currently prevalent in the sleep staging field. E-SCNN outperformed all other models on both datasets in terms of Acc and κ, indicating not only high classification accuracy but also strong agreement between predicted and true labels. In terms of overall performance, E-SCNN achieved the best accuracies of 84.9% and 85.5% on the SC and MASS datasets, respectively. Metrics such as κ and F1 score also reflected that E-SCNN performed better than or similarly to other methods, combining the advantages of machine learning and deep learning methods. E-SCNN obtained a κ of 84.3% on the SC dataset, which was 0.2% less than that of SeqSleepNet. On the MASS dataset, the κ of E-SCNN was 84.7%, which was consistent with the performance of AttnSleep. This finding is also supported by the results shown in Figure 7.

The evaluation of class-wise performance in automatic sleep staging, as presented in Table 4, revealed significant insights into the effectiveness of the E-SCNN model across various sleep stages. It was crucial to analyze the performance per class because each sleep stage had distinct characteristics and clinical relevance. E-SCNN achieved an impressive F1 score of 91.6% on the SC dataset, showcasing its exceptional capability in accurately identifying wake stages. This high score indicates that the model is adept at distinguishing wake stages from other sleep stages with minimal false negatives or positives. In the N1 stage, E-SCNN obtained an F1 score of 50.6% on the MASS dataset and 40.9% on the SC dataset, highlighting its ability to accurately classify the light sleep phase, which is often challenging due to its subtle differences relative to wakefulness and lighter NREM stages. The detailed class-wise analysis of E-SCNN’s performance provided a comprehensive view of its strengths and limitations across different sleep stages. In terms of class-wise performance, E-SCNN demonstrated exceptional precision in identifying wake stages, non-REM stages, and REM sleep, which is fundamental for both research and practical applications in sleep diagnostics and monitoring.

## 4. Discussion

The superior performance of E-SCNN in the automatic sleep staging task highlights its potential as a robust solution for sleep analysis. In this section, we delve into the reasons behind these findings and discuss their implications.

### 4.1. Effect of PCA

**Figure 8:**
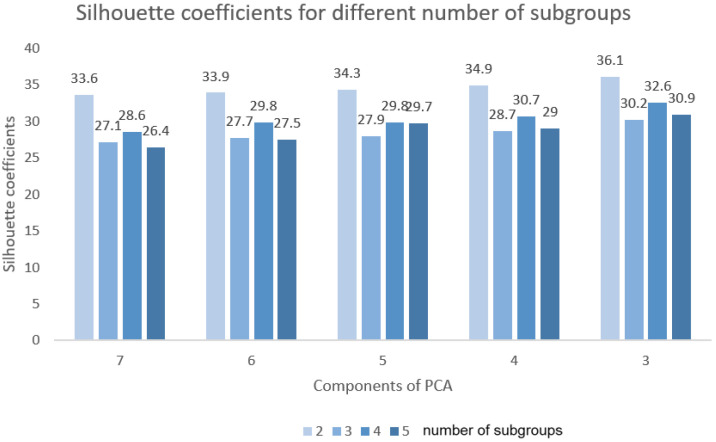
Trend of silhouette coefficients for different numbers of subgroups and components.

Figure 8 illustrates the trend of the silhouette coefficients relative to the number of principal components, as well as the number of subgroups. As the number of principal components decreased, the ROI gradually dropped. However, when the number of principal components was reduced from seven to five, the ROI decreased from 99.0% to 96.9%, indicating that the data dimensionality can be effectively reduced while maintaining a high level of information retention. The effectiveness of clustering was measured by the silhouette coefficient. The silhouette coefficient increased as the number of components decreased. This indicates that the original data contained a lot of noise or redundant information and that reducing the number of PCA components may help to remove this noise and redundancy. Therefore, PCA could make it easier for the clustering module to identify suitable cluster structures.

### 4.2. Existence and Impact of Interpersonal Differences

There may be differences in the sleep structure of different populations, and such differences are caused by a variety of factors. For example, children’s sleep outcomes are significantly different from those of adults, with a higher percentage of REM stage during sleep, with that percentage gradually decreasing with age. There are also variations in sleep structure between different subjects due to individual characteristics. These differences are one of the main reasons for the lack of precision in sleep staging. According to the results shown in Table 3, E-SCNN performed better than the SCNN and was more focused on sleep staging for individuals. The population division of a priori knowledge minimized within-group variation, reducing the perturbation of the model by a few outliers. Therefore, preliminary population segmentation through machine learning methods can effectively enhance the sleep stage classification of such individual variability.

### 4.3. Limitations

We need to point out some limitations of the experiment. First, the clustering module solely relied on conventional features such as power spectrum, fast waves, and slow waves, without incorporating the entropy and nonlinear features, which could potentially enhance the clustering process. Second, the proposed method improved or maintained a smooth performance for most individuals and showed performance degradation for certain individuals. This indicates that the stability of the clustering module needs to be improved. On the one hand, non-linear features can be added to further refine the pre-segmentation of the population, and individuals can be filtered based on clustering distances, with weights not applied to individuals beyond a specific distance. Finally, to explore the differences in the distribution of different characteristics within the population, this paper utilized two publicly available datasets without validation on the children’s dataset. The differences in the internal distribution of these two datasets are mainly in the areas of gender, age, and variations in individuals. The analysis presented in this paper revealed that the similarity of time-frequency characteristics in different populations had an impact on the results of sleep staging and that clustering can help to improve classification ability.

### 4.4. Future Works

There are several promising directions for the enhancement and expansion of this work. More interpretable features like age and gender can be employed within the clustering module, making the results more understandable and reliable. By incorporating features that are computationally efficient, the complexity of neural networks can be reduced, thereby forming an efficient ensemble model. Other supervised learning methods such as random forests can also be utilized as alternatives to the clustering module to explore the most effective ensemble approaches. Furthermore, there are fewer N1 stage samples than those for other stages, leading to biased predictions and reduced performance. Techniques need be explored to address potential issues related to imbalanced datasets, thereby improving the generalization of the model.

## 5. Conclusions

In this paper, a robust automatic sleep staging method that ensembles machine learning and deep learning techniques is proposed. It begins with the segmentation of the population using PCA and clustering. Then, an SCNN is employed to extract features from the signal. Finally, the clustering weights are incorporated with the features, yielding an individualized prediction of sleep stages. This scheme takes into account the variability of different individuals within the population and adopts the respective advantages of machine learning and deep learning in a comprehensive manner. Thus, it effectively leverages the interpretability of machine learning and the feature extraction power of deep learning and achieves a balance between generalization and precision, especially for outlier individuals.The improvements in accuracy suggest that this approach could lead to more reliable and efficient sleep analysis tools, ultimately contributing to better health outcomes.

## Figures and Tables

**Table 1:** Details of the two public datasets. SC and MASS represent the SC subset of the Sleep-EDF Expanded dataset and the SS3 subset of MASS dataset, respectively.

Dataset	Subjects	EEG Channel	Stages					
			Wake	N1	N2	N3	REM	Total
SC	78	FPz-Cz	65,951	21,522	69,132	13,039	25,835	195,479
MASS	62	C4-A1	6442	4839	29,802	7653	10,581	59,317

**Table 2:** Silhouette coefficients for different numbers of subgroups and components.

Component	Retention of Information	Silhouette Coefficients			
		2	3	4	5
7	99.0	33.6	27.1	28.6	26.4
6	98.1	33.9	27.7	29.8	27.5
5	96.9	34.3	27.9	29.8	29.7
4	94.6	34.9	28.7	30.7	29.0
3	91.6	36.1	30.2	32.6	30.9

**Table 3:** Comparison of overall effects and class-wise cases between E-SCNN and SCNN. The bold values represent the best result in the corresponding datasets.

Dataset	Model	Overall Metrics					Class-Wise Performance (F1)				
		Acc	κ	F1	Sens	Spec	Wake	N1	N2	N3	REM
SC	SCNN	84.4	83.8	78.0	78.3	95.8	91.5	40.6	86.9	83.9	87.0
	E-SCNN	**84.9**	**84.3**	**78.1**	**78.5**	**96.0**	**91.6**	**40.9**	**87.0**	**84.2**	**87.0**
MASS	SCNN	85.2	84.6	79.4	79.5	95.8	89.1	50.3	**89.8**	82.3	**85.3**
	E-SCNN	**85.5**	**84.7**	**79.5**	**79.7**	**95.8**	**89.6**	**50.6**	89.7	**82.5**	85.2

**Table 4:** Results comparison with state-of-the-art methods on two datasets. The bold values represent the best result in the corresponding datasets.

	Model	Overall Metrics					Class-Wise Performance (F1)				
		Acc	κ	F1	Sens	Spec	Wake	N1	N2	N3	REM
SC	E-SCNN	**84.9**	84.3	78.1	78.5	**96.0**	**91.6**	40.9	87.0	84.2	87.0
	DeepSleepNet [30]	84.5	83.9	77.3	78.5	94.2	86.3	46.8	86.2	84.5	83.6
	SeqSleepNet [31]	84.6	**84.5**	78.3	**79.9**	94.4	89.0	**58.6**	**89.2**	80.2	**88.5**
	THNN [47]	84.7	84.0	78.1	78.5	95.0	90.6	46.3	87.3	81.9	81.3
	SimpleSleepNet [48]	84.0	83.4	**79.7**	78.2	94.7	88.7	60.0	88.3	74.1	87.3
	AttnSleep [49]	83.3	82.9	76.8	77.2	94.0	91.0	42.2	86.1	81.6	75.3
MASS	E-SCNN	**85.5**	**84.7**	79.5	79.7	**95.8**	89.6	50.6	**89.7**	82.5	85.2
	DeepSleepNet [30]	85.0	84.2	78.0	77.0	94.1	82.3	48.2	89.6	82.0	87.9
	SeqSleepNet [31]	84.8	84.1	**80.2**	**80.0**	95.1	84.6	54.3	89.6	84.6	77.2
	THNN [47]	81.3	79.9	68.7	75.6	93.8	**90.7**	52.2	80.8	77.9	80.1
	SimpleSleepNet [48]	83.7	83.1	77.3	79.9	94.3	88.5	47.2	81.7	81.2	**88.5**
	AttnSleep [49]	85.2	84.7	78.0	77.5	95.5	86.0	**55.6**	87.2	**90.2**	79.5

## Data Availability

The datasets can be found at https://physionet.org/content/sleep-edfx/1.0.0/ and http://ceams-carsm.ca/en/MASS/, accessed on 21 August 2024.
